# Immunoassay with Novel Paired Antibodies for Detection of Lipoarabinomannan in the Pleural Fluid and Plasma of Patients with Tuberculous Pleurisy

**DOI:** 10.3390/microorganisms11092259

**Published:** 2023-09-08

**Authors:** Zhuohong Yan, Jinghui Wang, Yu Pang, Xiaojue Wang, Ling Yi, Panjian Wei, Hongyun Ruan, Meng Gu, Hongtao Zhang, Xinting Yang

**Affiliations:** 1 Department of Central Laboratory, Beijing Chest Hospital, Capital Medical University, Beijing Tuberculosis and Thoracic Tumor Research Institute, Beijing 101149, China; 2 Department of Medical Oncology, Beijing Chest Hospital, Capital Medical University, Beijing Tuberculosis and Thoracic Tumor Research Institute, Beijing 101149, China; 3 Department of Bacteriology and Immunology, Beijing Key Laboratory on Drug-Resistant Tuberculosis Research, Beijing Chest Hospital, Capital Medical University, Beijing Tuberculosis and Thoracic Tumor Research Institute, Beijing 101149, China; 4 Department of Tuberculosis, Beijing Chest Hospital, Capital Medical University, Beijing 101149, China

**Keywords:** tuberculous pleurisy, lipoarabinomannan, pleural fluid, sandwich ELISA, plasma

## Abstract

Tuberculous pleurisy (TP) is one of the most common forms of extrapulmonary tuberculosis, but its diagnosis is challenging. Lipoarabinomannan (LAM) antigen is a biomarker for *Mycobacterium tuberculosis* (Mtb) infection. LAM detection has potential as an auxiliary diagnostic method for TP. We have successfully generated five rabbit anti-LAM monoclonal antibodies (BJRbL01, BJRbL03, BJRbL20, BJRbL52, and BJRbL76). Here, anti-LAM antibodies were tested to detect LAM in the pleural fluid and plasma of patients with TP by sandwich enzyme-linked immunosorbent assays (ELISAs). The results revealed that all of the anti-LAM antibodies were successfully used as capture and detection antibodies in sandwich ELISAs. The BJRbL01/BJRbL01-Bio pair showed better performance than the other antibody pairs for detecting mycobacterial clinical isolates and had a limit of detection of 62.5 pg/mL for purified LAM. LAM levels were significantly higher in the pleural fluid and plasma of patients with TP than in those of patients with malignant pleural effusion or the plasma of non-TB, and LAM levels in the pleural fluid and plasma were positively correlated. Moreover, LAM levels in the pleural fluid sample were significantly higher in confirmed TP patients than in clinically diagnosed TP patients. Our studies provide novel LAM detection choices in the pleural fluid and plasma of TP patients and indicate that LAM detection assay has an auxiliary diagnostic value for TP, which may help to improve the diagnosis of TP.

## 1. Introduction

Tuberculosis (TB) is caused by *Mycobacterium tuberculosis* (Mtb) and was the world’s leading cause of death from a single infectious microorganism until the pandemic of coronavirus (COVID-19) [1]. Despite TB being both preventable and curable, an estimated 10.6 million people fell ill with TB in 2021, which is an increase of 4.5% from the 10.1 million in 2020 [1]. The TB incidence rate is estimated to have increased by 3.6% between 2020 and 2021. In 2021, there were an estimated 1.6 million people who died of this disease [1]. 

TB primarily affects the lungs, namely, pulmonary tuberculosis (PTB), and also frequently occurs in extrapulmonary sites, such as the pleura, lymph nodes, and bones, resulting in extrapulmonary tuberculosis (EPTB). EPTB represents approximately 15–20% of all TB worldwide [2,3]. Diagnosis of EPTB is challenging due to its obscure paucibacillary nature, location, broad spectrum of clinical manifestations, and the frequent presence of undetected lesions at multiple body sites that can cause occult disease [4,5,6].

China has the third highest number of TB cases worldwide, accounting for 7.4% of global TB incidence [1]. In China, 24.6% of TB patients has EPTB according to a recent national survey [7]. From January 2008 to December 2017, 33.4% of hospitalized TB patients in Beijing Chest Hospital had EPTB [8]. Based on a large-scale multi-center investigation, the most common EPTB lesion in China was tuberculous pleurisy (TP, 49.8%) [4]. Two-thirds of people with simple TP will progress to pulmonary TB within 2 years if left untreated [9]. Therefore, the diagnosis of TP should not be neglected.

Pleural effusions of TP are paucibacillary, so usual tests for mycobacteriologic confirmation have low yields [10]. The yield of the mycobacterial culture of pleural fluid depends on the culture medium used [9]. The sensitivity of solid culture media such as Lowenstein–Jensen medium is <30% [11]. The sensitivity of liquid culture media such as the BACTEC-MGIT semi-automated system (Becton-Dickinson, Franklin Lakes, NJ, USA) is up to 70% [9]. Nucleic acid amplification tests (NAAT), like the GeneXpert platform, are highly specific to TB but have relatively poor sensitivity in pleural fluid and pleural tissue. The overall sensitivity of NAAT for tuberculous pleural effusion ranges between 28% and 81% in pleural fluid and is 90% in pleural tissue, with a specificity between 90% and 100% [9,12]. Adenosine deaminase and interferon-gamma are the most notable surrogate biomarkers of TB used in the diagnosis of TP.

Pleural effusions arise in TP patients because of an effusive T-helper cell type 1 inflammatory response to Mtb antigen in the pleural space, which upsets the balance of Starling forces that determine pleural fluid formation/resorption [9]. Mtb antigens in the pleural fluid may serve as biomarkers of TP. Lipoarabinomannan (LAM) is a specific component of the cell wall of Mtb [13]. During Mtb infection, LAM exists in a variety of body fluids; therefore, it can be an ideal candidate biomarker for detecting Mtb [14]. Detecting LAM in urine by anti-LAM antibodies fills a gap in the rapid diagnosis of TB. Alere Determine TB LAM Ag (AlereLAM; Abbott, Palatine, IL, USA) has been recommended by the World Health Organization for TB diagnosis in HIV-positive patients [15]. AlereLAM lacks high sensitivity in all TB patients. In recent years, FujiLAM (Fujifilm SILVAMP TB LAM Fujifilm, Tokyo, Japan) promises greater diagnostic sensitivity compared to the AlereLAM in HIV-positive patients [16,17] and may have a diagnostic value in HIV-negative patients [18,19]. FujiLAM uses silver-amplified immunochromatography on the lateral flow strip, and its analytical sensitivity is 30 times higher than that of AlereLAM [20].

The detection of LAM in pleural fluid may be an auxiliary diagnosis assay for TP. We have successfully generated five rabbit anti-LAM monoclonal antibodies (mAbs) with high sensitivity and specificity for detecting LAM in indirect ELISA assays [21]. In this study, we aimed to screen the suitable paired antibodies to detect LAM in pleural fluid and plasma samples from patients with TP by sandwich ELISA and preliminarily evaluate its application value in the auxiliary diagnosis of TP.

## 2. Materials and Methods

### 2.1. Anti-LAM Antibodies

Rabbit anti-LAM mAbs BJRbL01, BJRbL03, BJRbL20, BJRbL52, and BJRbL76 were screened and used in this study [21].

### 2.2. Ethics Statement

This study was approved by the Ethics Committee of Beijing Chest Hospital, Capital Medical University, Beijing, China. Samples from enrolled subjects were collected after receiving written informed consent from the participants. 

### 2.3. Bacterial Strains 

Inactivated bacterial culture supernatant from 23 mycobacterial species, including *M.tb H37Rv*, *M. bovis*, slowly growing non-tuberculous mycobacteria (NTM) strains (*n* = 9), rapidly growing NTM strains (*n* = 12), 30 Mtb isolates, and 10 *M. abscessus* (MA) isolates, were used to screen the best-paired anti-LAM mAbs. Slowly growing NTM included *M. kansasii*, *M. marinum*, *M. scrofulaceum*, *M. gordonae*, *M. xenopi*, *M. avium*, *M. intracellulare*, *M. gastri*, and *M. malmoense*; rapidly growing NTM strains included *M. smegmatis*, *M. fortuitum*, *M. aurum*, *M. neoaurum*, *M. abscessus*, *M. parafortuitum*, *M. salmoniphilum*, *M. nonchromogenicum*, *M. vaccae*, *M. phlei*, *M. confluentis*, and *M. gilvum*. The inactivated supernatant of *Streptococcus pneumoniae*, *Staphylococcus aureus*, *Pseudomonas aeruginosa*, and *Haemophilus influenzae* were used to test the specificities of the anti-LAM Abs. The supernatants were stored at −80 °C until use. 

### 2.4. Study Design and Participants

Pleural fluid specimens were prospectively collected in a cohort study to identify the appropriate algorithm for the care of patients with TP [22,23]. Adults with suspected TP were enrolled consecutively in four hospitals (Beijing Chest Hospital, Beijing Chao Yang Hospital, Beijing Geriatric Hospital, and Beijing Hospital) in China from July 2015 to January 2018. Enrolled patients had not been treated with any anti-TB drugs in the past 6 months. All of the patients were followed up for a minimum of 12 months. Each pleural fluid specimen was subjected to smear microscopy, culture, Xpert, and routine biochemical examinations, simultaneously. Plasma specimens from partial patients were also collected. Aliquot pleural fluid and plasma samples were stored in the Beijing Bio-Bank of Clinical Resources on Tuberculosis (Beijing Chest Hospital) at −80 °C until use. 

### 2.5. Patient Categories

Patients were divided into three groups (TP, MPE (malignant pleural effusion), and non-TB group) according to the composite reference standard. The diagnostic criteria for TP were according to WS 288-2017 Tuberculosis Diagnosis Guidelines [24]. The TP group (*n* = 160) included laboratory-confirmed TP (*n* = 88) and clinically diagnosed TP patients (*n* = 72). Confirmed TB patients were represented by a positive outcome from at least one culture- and/or Xpert-positive pleural fluid sample for MTB and/or pleural biopsy tissue; clinically diagnosed TP patients were without any positive laboratory evidence by culture and Xpert but had been diagnosed with active pleural TB by a physician according to clinical findings, thoracoscopic reports, radiologic imaging, and 12 months of follow-up outcome since the date of enrollment. MPE patients (*n* = 50) were characterized by the presence of malignant cells in the pleural fluid. Non-TB subjects showed no clinical signs or symptoms of TB and had no history of TB. All participants were HIV-negative and had no history of NTM disease. 

### 2.6. ELISA

Sandwich ELISA was performed to analyze the binding epitopes of anti-LAM antibodies and test the sensitivities of the selected paired antibodies. Capture antibodies were coated at 1 μg/mL to polystyrene plates at 4 °C overnight. The next day, the plates were blocked with 5% skimmed milk after washing and then incubated with 1 µg/mL or serial dilutions of purified *M.tb H37Rv* LAM (BEI Resources, NR-14848), 1:50 dilution of heat-killed diluted suspensions of Mtb clinical isolates, and 100 µL of pleural fluid or plasma samples at 4 °C overnight. After five washes, the plates were incubated with 1 μg/mL of biotin-labeled anti-LAM Abs at 37 °C for 2 h and then incubated with horseradish peroxidase (HRP)-coupled streptavidin at a 1:5000 dilution for 1 h at 37 °C after washing. A final incubation with TMB (tetramethylbenzidine) substrate solution for 30 min at 37 °C was used for Ab antigen detection. The optical density at 450 nm (OD_450_) was measured after adding a stop solution.

Indirect ELISA was used to detect the specificities of anti-LAM mAbs to common pneumonia-causing pathogenic bacteria. Microplate wells were coated with 1:100 dilution of heat-killed diluted supernatant of pathogenic bacteria, blocked with 5% skimmed milk, and then incubated with 1 µg/mL of each rabbit anti-LAM mAb at 37 °C for 2 h. After five washes, the plates were incubated with HRP-coupled goat anti-rabbit IgG (H + L) Ab at a 1:5000 dilution for 1 h at 37 °C. OD_450_ was measured after adding a stop solution. 

### 2.7. Statistics

Statistical analysis was conducted using GraphPad Prism 7 software (GraphPad Software Inc., San Diego, CA, USA). Statistical analysis was performed using a Mann–Whitney test. ROC curves were used to evaluate the diagnostic value of LAM in patients with TP. Cut-off values were either determined using an ELISA based on data from MPE or the non-TB group and defined as the mean OD_450_ value + 2 × standard deviations (SD) or were determined from ROC curves as the optimal cut-off values when the Youden index (sensitivity + specificity − 1) was at the maximum. Differences were considered significant when *p* < 0.05. 

## 3. Results

### 3.1. Screening of Paired Anti-LAM Antibodies by Sandwich ELISA

**Figure 1:**
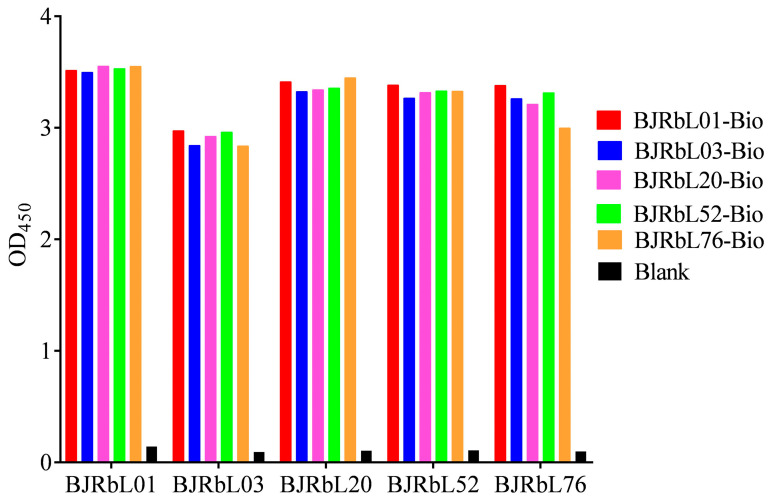
Screening paired anti-LAM antibodies in sandwich ELISAs. Unlabeled and biotin-labeled anti-LAM antibodies were used as capture and detection antibodies to detect purified LAM from *M.tb H37Rv*.

We generated the rabbit anti-LAM mAbs, including BJRbL01, BJRbL03, BJRbL20, BJRbL52, and BJRbL76, in our previous study. In this study, we first evaluated whether these antibodies could identify purified LAM in a sandwich ELISA. We found that each of our anti-LAM mAbs was suitable for use as a capture antibody or a detection antibody for LAM in a sandwich ELISA (Figure 1).

### 3.2. Sensitivity of the Paired Anti-LAM mAbs for Purified LAM

**Figure 2:**
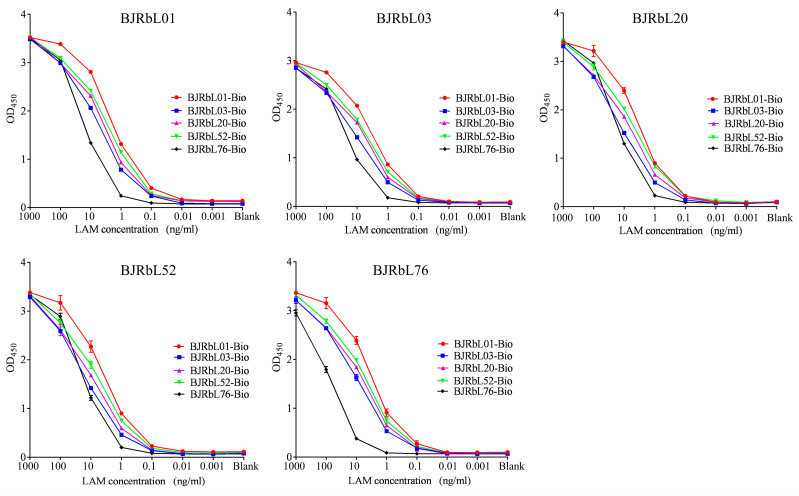
Sensitivities of paired anti-LAM antibodies for purified LAM by sandwich ELISA. Each anti-LAM antibody was used as a capture antibody and a detection antibody in the sandwich assay. Purified LAM from *M.tb H37Rv* was used as the detection antigen.

Next, the sensitivities of the paired anti-LAM Abs were determined by sandwich ELISA. Each combination of antibodies using BJRbL01, BJRbL03, BJRbL20, or BJRbL76 as a capture antibody and BJRbL01-Bio, BJRbL20-Bio, or BJRbL52-Bio as a detection antibody had a sensitivity of 0.1 ng/mL. Antibody pairs using BJRbL52 as a capture antibody and BJRbL03-Bio or BJRbL76-Bio as detection antibodies had a moderate sensitivity in the range of 1.0 ng/mL. Antibody pairs using BJRbL76 as a capture antibody and BJRbL76-Bio as detection antibodies had a relatively low sensitivity in the range of 10.0 ng/mL (Figure 2 and Table 1). 

### 3.3. Reactivity of the Paired Anti-LAM mAbs to Mycobacterial Species

**Figure 3:**
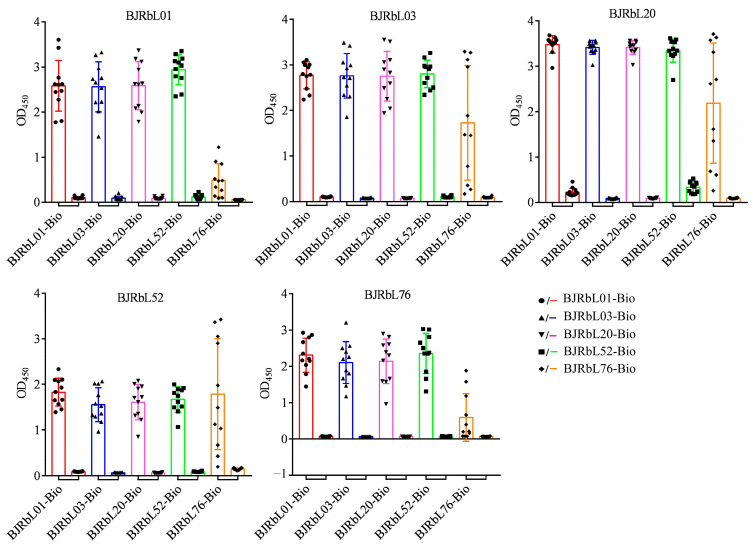
Reactivity of the paired anti-LAM mAbs to multiple mycobacterial species in sandwich ELISAs. Eleven slow-growing mycobacterial species (left) and twelve rapid-growing NTM strains (right) were tested for each antibody combination. Slowly growing mycobacterium species included *M.tb H37Rv*, *M. bovis*, *M. kansasii*, *M. marinum*, *M. scrofulaceum*, *M. gordonae*, *M. xenopi*, *M. avium*, *M. intracellulare*, *M. gastri*, and *M. malmoense*; rapidly growing NTM strains included *M. smegmatis*, *M. fortuitum*, *M. aurum*, *M. neoaurum*, *M. abscessus*, *M. parafortuitum*, *M. salmoniphilum*, *M. nonchromogenicum*, *M. vaccae*, *M. phlei*, *M. confluentis*, and *M. gilvum*.

We further evaluated whether the paired anti-LAM mAbs could be used to detect mycobacterial species by sandwich ELISA. All paired anti-LAM mAbs reacted with inactivated supernatants from cultures of *M.tb H37Rv*, *M. bovis*, and slow-growing NTM strains (*n* = 9), but not with supernatants from cultures of rapid-growing NTM strains (*n* = 12) (Figure 3). In assays with BJRbL01, BJRbL03, BJRbL20, BJRbL52, or BJRbL76 as capture antibodies and BJRbL01-Bio, BJRbL03-Bio, BJRbL20-Bio, or BJRbL52-Bio as detection antibodies the sensitivities and specificities of all combinations were both 100%. When used as a detection antibody, BJRbL76-Bio had a lower sensitivity (81.8% for BJRbL76/BJRbL76-Bio, 90.0% for BJRbL03/BJRbL76-Bio and BJRbL52/BJRbL76-Bio, and 100% for BJRbL01/BJRbL76-Bio and BJRbL20/BJRbL76-Bio) and mean OD_450_ value than the other Ab pairs.

### 3.4. Reactivity of the Paired Anti-LAM mAbs to Mycobacterial Clinical Isolates

**Figure 4:**
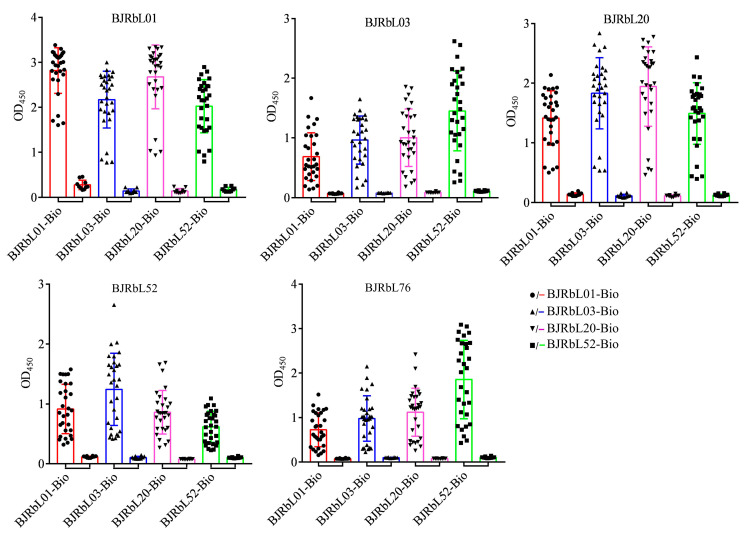
Reactivity of the paired anti-LAM mAbs to multiple mycobacterial clinical isolates in sandwich ELISAs. Thirty *M. tuberculosis* isolates (left) and ten *M. abscessus isolates* (right) were used to evaluate the performance of each anti-LAM antibody pair in sandwich ELISAs.

We next investigated whether the paired anti-LAM mAbs could be used to detect mycobacterial clinical isolates by sandwich ELISA. Thirty Mtb isolates and ten *M. abscessus* (MA) isolates were used in this assay. We found that the paired anti-LAM mAbs could identify all Mtb isolates but did not react with MA isolates (Figure 4). The pair BJRbL01/BJRbL01-Bio had the highest mean OD_450_ value, and BJRbL01/BJRbL20-Bio had the second highest (Figure 4).

### 3.5. Reactivity of Anti-LAM mAbs to Common Pneumonia-Causing Pathogenic Bacteria

**Figure 5:**
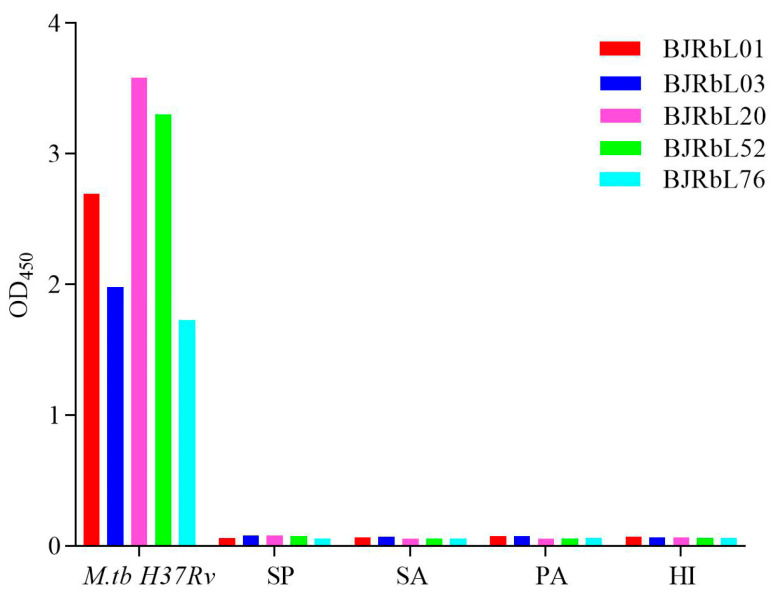
Reactivity of the anti-LAM mAbs to common pneumonia-causing pathogenic bacteria. Reactivity of the anti-LAM mAbs to the inactivated supernatants of pathogenic bacteria that commonly cause pneumonia as measured by indirect ELISA. SP: *Streptococcus pneumoniae;* SA: *Staphylococcus aureus*; PA: *Pseudomonas aeruginosa*; HI: *Haemophilus influenzae*.

TP must usually be differentiated from pneumonia caused by other pathogenic bacterial infections in the clinic. We analyzed the specificity of our anti-LAM antibodies to common pathogenic bacteria that cause pleural effusion and pleural empyema, including *Streptococcus pneumoniae*, *Staphylococcus aureus*, *Pseudomonas aeruginosa*, and *Haemophilus influenzae*. All of our anti-LAM antibodies did not react with these bacteria (Figure 5). 

### 3.6. LAM Detection in the Pleural Fluid of Patients with TP

**Figure 6:**
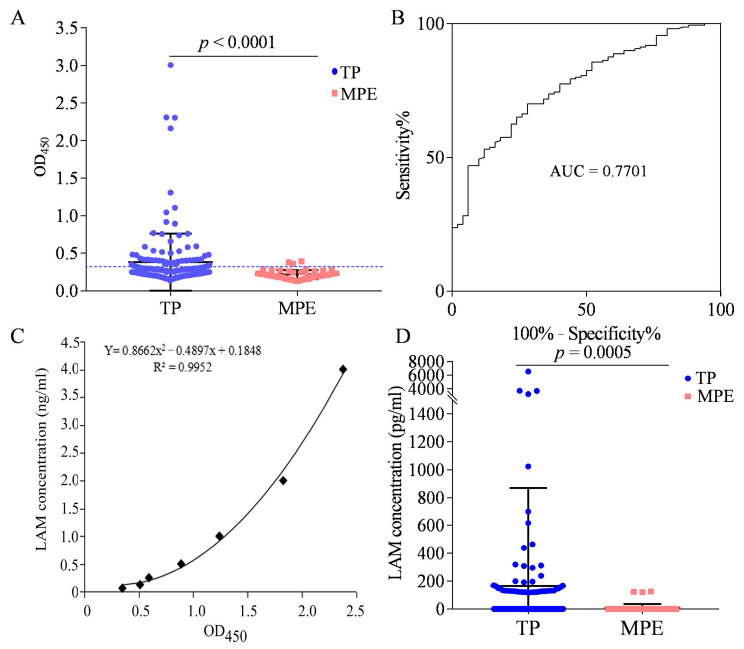
LAM detection in the pleural fluid of patients with tuberculous pleurisy (TP). (**A**) LAM detection in the pleural fluid of TP patients (*n* = 160) and malignant pleural effusion (MPE) samples (*n* = 50) by sandwich ELISA. Horizontal lines indicate the cut-off based on the mean value from the MPE group plus two standard deviations. (**B**) The receiver operating characteristic (ROC) curve analysis for the evaluation of LAM detection capacity in TP patients. (**C**) The purified LAM standard curve as detected with the BJRbL01 and BJRbL01-Bio antibodies by sandwich ELISA. (**D**) The concentration of LAM in the pleural fluid. Differences were assessed by a Mann–Whitney test.

We used BJRbL01 and BJRbL01-Bio to detect LAM in the pleural fluid of patients with TP (*n* = 160). MPE samples (*n* = 50) were used as negative controls. The characteristics of the study participants are summarized in Table 2. We found that LAM levels were significantly higher in the TP group than in the MPE group (Figure 6A). The ROC curves were used to evaluate the auxiliary diagnostic value of LAM detection for TP. The area under the ROC curves (AUCs) was 0.7701 (95% CI, 0.7007–0.8394) (Figure 6B). When the cut-off value was determined using the Youden index, the sensitivity was 70.0% (112/160; 95% CI, 62.26–76.98%) and the specificity was 72.0% (36/50; 95% CI, 57.51–83.77%). When the cut-off value was determined as the mean OD_450_ value + 2 × SD of the MPE group, the detection sensitivity was 30.6% (49/160; 95% CI, 23.59–38.39%) and the specificity was 94.0% (47/50; 95% CI, 83.45–98.75%). 

The standard curve of purified LAM detected by BJRbL01 and BJRbL01-Bio antibodies is shown in Figure 6C, and the limit of detection (LOD) for LAM was 62.5 pg/mL based on the cut-off value of the ELISA assay. Using this standard curve, we calculated the LAM concentrations in the pleural fluid samples (Figure 6D). Samples with OD_450_ values below the cut-off value (mean OD_450_ value + 2 × SD of the MPE group) were considered to have no detectable levels of LAM (recorded as 0). Among the 49 positive samples (with OD_450_ values above the cut-off value), the concentration of LAM ranged from 118.0 to 6530.9 pg/mL, and four samples had LAM concentrations higher than 3000 pg/mL (Figure 6D). There were three positive samples in the MPE group, with LAM concentrations ranging from 120.7 to 126.0 pg/mL. 

### 3.7. LAM Antigen Detection in the Plasma of Patients with TP

**Figure 7:**
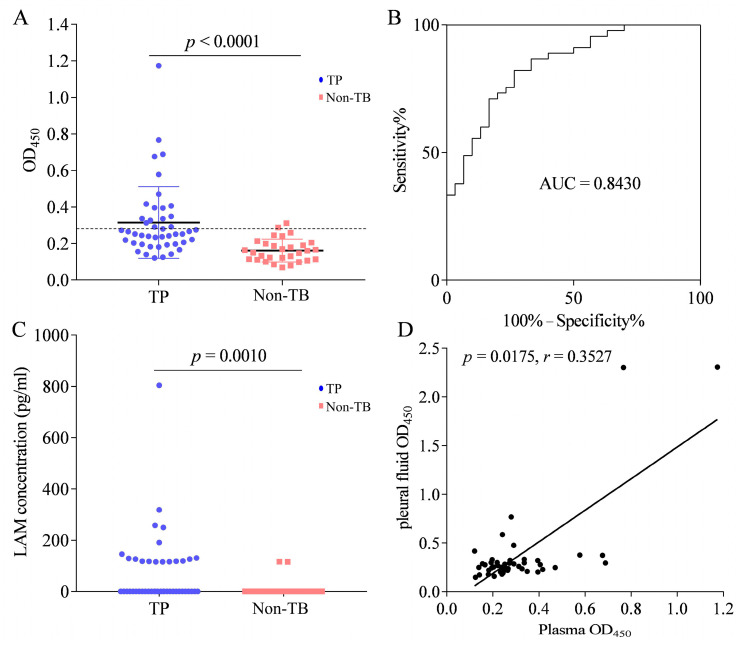
LAM detection in the plasma of patients with tuberculous pleurisy (TP). (**A**) LAM detection in the plasma of patients with TP (*n* = 45) and non-TB (*n* = 30) by sandwich ELISA. Horizontal lines indicate the cut-off based on the mean value from non-TB plus two standard deviations. (**B**) The receiver operating characteristic (ROC) curve analysis for the evaluation of LAM detection capacity in TP patients. (**C**) The concentration of LAM in the plasma. (**D**) The correlation analysis of LAM detection in the pleural fluid and plasma of TP patients. Differences were assessed by a Mann–Whitney test.

Of the 160 TP patients, plasma was collected from 45 patients. We found that plasma levels of LAM were significantly higher in the TP group than in the non-TB group (Figure 7A), with an AUC of 0.8430 (95% CI, 0.7546–0.9313) (Figure 7B). When the cut-off value was determined using the Youden index, the sensitivity was 82.2% (37/45; 95% CI, 67.95–92.0%) and the specificity was 73.3% (22/30; 95% CI, 54.11–87.72%). When the cut-off value was determined as the mean OD_450_ value + 2 × SD of the non-TB, the detection sensitivity was 37.8% (17/45; 95% CI, 23.77–53.46%) and the specificity was 93.3% (28/30; 95% CI, 77.93–99.18%). Among the 17 positive samples, the concentration of LAM ranged from 115.6 to 803.8 pg/mL (Figure 7C). Furthermore, we analyzed the relationship between LAM levels in the pleural fluid and plasma and found a significant positive correlation (*p* = 0.0175, r = 0.3527) (Figure 7D).

### 3.8. Characteristics of LAM Level in Confirmed and Clinically Diagnosed TP Patients

**Figure 8:**
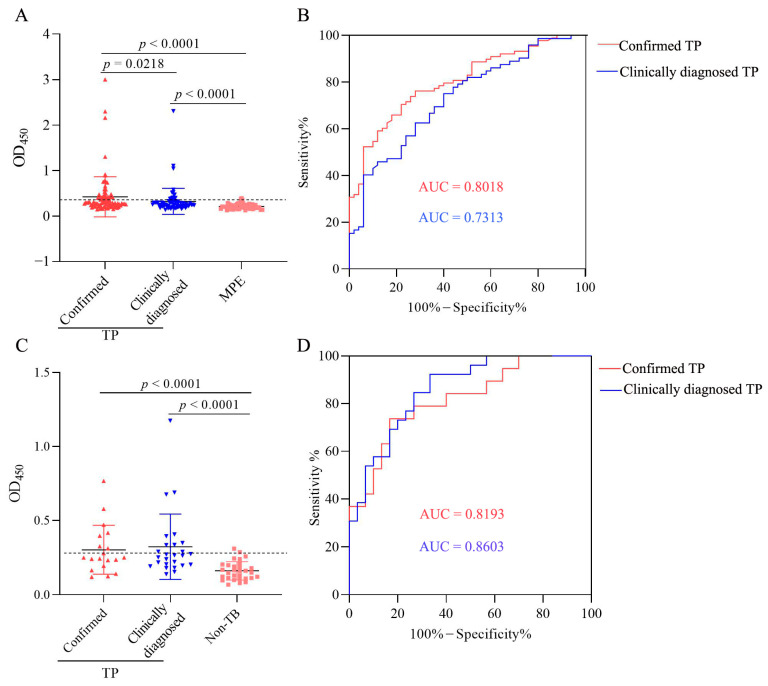
Analysis of LAM level in pleural fluid and plasma in patients with confirmed and clinically diagnosed tuberculous pleurisy (TP). (**A**) LAM level detection in the pleural fluid of patients with confirmed TP (*n* = 88), clinically diagnosed TP (*n* = 72), and malignant pleural effusion (MPE, *n* = 50) by sandwich ELISA. (**B**) The receiver operating characteristic (ROC) curve analysis for the evaluation of pleural fluid LAM detection capacity in the two TP subgroups. (**C**) LAM level detection in the plasma of subjects with confirmed TP (*n* = 19), clinically diagnosed TP (*n* = 26), and non-tuberculosis (non-TB, *n* = 30) by sandwich ELISA. (**D**) The ROC curve analysis for the evaluation of plasma LAM detection capacity in the two TP subgroups. Horizontal lines indicate the cut-off based on the mean value from MPE or non-TB plus two standard deviations, respectively. Differences were assessed by a Mann–Whitney test.

We further compared the LAM level in pleural fluid and plasma between confirmed TP and clinically diagnosed TP subgroups. We found that LAM levels in the pleural fluid were significantly higher in confirmed TP (*n* = 88) and clinically diagnosed TP (*n* = 72) subgroups than in the MPE group (*n* = 50), and LAM levels were significantly higher in confirmed TP than in the clinically diagnosed TP subgroup (Figure 8A). The AUCs of pleural fluid LAM assay were 0.8018 (95% CI, 0.7294–0.8742) and 0.7313 (95% CI, 0.6424–0.8201) for confirmed TP and clinically diagnosed TP subgroups (Figure 8B), respectively. When the cut-off value was determined using the Youden index, the sensitivities were 70.5% (62/88; 95% CI, 60.23–78.97%) and 75.0% (54/72; 95% CI, 63.91–83.56%), and the specificities were 78.0% (39/50; 95% CI, 64.76–87.25%) and 60.0% (30/50; 95% CI, 46.18–72.39%) for confirmed and clinically diagnosed TP patients, respectively. When the cut-off value was determined as the mean OD_450_ value + 2 × SD of the MPE group, the sensitivities were 39.8% (35/88; 95% CI, 30.18–50.22%) and 19.4% (14/72; 95% CI, 11.95–30.03%) for confirmed and clinically diagnosed TP patients, respectively.

The LAM levels in plasma were significantly higher in the confirmed TP (*n* = 19) and clinically diagnosed TP (*n* = 26) subgroups than in the non-TB group (*n* = 30) and showed no statistical difference between the two subgroups (Figure 8C). The AUCs of plasma LAM assay were 0.8193 (95% CI, 0.6972–0.9414) and 0.8603 (95% CI, 0.7666–0.9539) for confirmed TP and clinically diagnosed TP subgroups (Figure 8D), respectively. When the cut-off value was determined using the Youden index, the sensitivities were 73.7% (14/19; 95% CI, 51.21–88.19%) and 92.3% (24/26; 95% CI, 75.86–98.63%), and the specificities were 83.3% (25/30; 95% CI, 66.44–92.66%) and 60.0% (18/30; 95% CI, 42.32–75.41%) for the confirmed and clinically diagnosed TP patients, respectively. When the cut-off value was determined as the mean OD_450_ value + 2 × SD of the non-TB, the sensitivities were 36.8% (7/19; 95% CI, 19.15–58.96%) and 38.5% (10/26; 95% CI, 22.43–57.47%) for the confirmed and clinically diagnosed TP patients, respectively.

## 4. Discussion

LAM is a mycobacterial cell wall lipopolysaccharide and virulence factor. Soluble LAM released from bacterial and infected cells is an important immunodiagnostic target for Mtb infection and activation. Plasma and urine levels of LAM are the most studied TB biomarkers [25,26,27]. Three types of LAM have been described: mannose-capped LAM (ManLAM), phospho-myo-inositol-capped LAM (PILAM), and non-capped LAM (AraLAM) [28]. Present in all members of the Mtb complex and in other pathogenic Mycobacterium strains [29], ManLAM facilitates Mtb pathogenesis and intracellular trafficking [30]. ManLAM consists of four structural domains: a phosphatidyl-myo-inositol anchor, a mannan core, an arabinan domain, and different capping motifs that contribute to species and strain diversity [29,31,32].

In this study, we found that our anti-LAM mAbs were suitable for use as a capture or detection antibody to analyze the LAM levels of purified ManLAM, *M.tb H37Rv*, *M. bovis*, multiple slow-growing NTM strains, and mycobacterial clinical isolates by sandwich ELISA. It is worth noting that LAM detection alone could not discriminate between Mtb and the slow-growing NTM, such as *M. avium* and *M. intracellulare*. More importantly, none of the antibody pairs reacted with rapid-growing NTM strains. Interestingly, the same anti-LAM mAb was suitable for use as a capture antibody or a detection antibody to detect ManLAM in a sandwich ELISA, with BJRbL01/BJRbL01-Bio showing the best performance for testing Mtb clinical isolates in our study. This may be related to the unique structure of ManLAM, which contains multiple tandem repeat sites, such as Ara4 (β-Araf-(1→2)-α-Araf-(1→5)-α-Araf-(1→5)-α-Araf) and Ara6 of LAM [31]. Several LAM epitopes have been reported, including long-chain Ara, Ara4/Ara6, Man only, Man2, Man3, Ara6 ± cap, Ara4/Ara6 ± Man1, Ara6 ± Man1, Mancap + MTX, and Mancap ± MTX [31]. Previously characterized rabbit anti-LAM mAbs mainly recognize Ara4/Ara6 ± Man1, Ara6 ± Man1, Mancap + MTX, and Mancap ± MTX epitopes [31].

Our antibodies showed good specificity for Mtb as they did not react with common pathogenic bacteria that cause pleural effusion in the clinic, such as *S. pneumoniae*, *S. aureus*, *P. aeruginosa*, and *H. influenzae*. Prior to the introduction of antibiotics, most pleural infections were caused by *S. pneumonia* (60–70%), followed by *S. pyogenes* and *S. aureus* [33,34]. However, *S. aureus* was the most frequently isolated organism (20.7%) after the introduction of antibiotics based on a recent systematic review by Hassan and colleagues that examined 6202 bacterial isolates in adults [35]. These findings indicated that our anti-LAM antibodies may be used to specifically detect Mtb in clinical specimens.

ManLAM is heterogeneous even within the Mtb complex group, which comprises Mtb, *M. bovis*, *M. microti*, and *M. africanum*. Indeed, ManLAM can vary, primarily its degree of terminal mannose capping, by 40% to 70% [31]. Moreover, enzymes in body fluids and tissues can impact LAM composition [31,36]. Few studies have been published about LAM detection in the pleural fluid. Mustafa et al. have previously tried to detect LAM in pleural fluid mononuclear cells from tuberculous pleural effusions [37]. In this study, we found that LAM levels were significantly higher in the pleural fluid and plasma of patients with TP than in those of patients with MPE or non-TB. MPE is one of the most common causes of exudative and unilateral pleural effusion [38]. Lung and breast cancer account for 50–65% of all malignant pleural effusions [39]. Our results preliminarily indicated that our paired anti-LAM antibodies could specifically detect LAM in pleural fluid and plasma specimens.

In addition, LAM levels in pleural fluid and plasma were positively correlated in patients with TP, such that the patients with the highest levels of LAM in their pleural fluid also had the highest LAM levels in their plasma, and LAM concentrations in the pleural fluid were higher than those in plasma. LAM detection in the plasma appeared to be better than that in the pleural fluid (AUC: 0.8430 vs. 0.7701; sensitivity: 82.2% vs. 70%; specificity: 73.3% vs. 72%), although this should be verified using larger sample sizes. Studies on LAM in pleural fluid and plasma in patients with TP are scarce, and our study provided preliminary proof of this. Currently, LAM detection sensitivities vary from 1 fg/mL to 3–28 ng/mL [14], which may be related not only to the detection methods but also to the different LAM standards used. The LOD of LAM detection was 62.5 pg/mL in our study, and this was acceptable when compared with the reported sensitivities (1 fg/mL to 3–28 ng/mL) [14].

LAM levels in pleural fluid and plasma were significantly higher in confirmed and clinically diagnosed TP subgroups than in MPE patients or non-TB subjects. Moreover, LAM levels in the pleural fluid were significantly higher in confirmed TP patients than in clinically diagnosed TP patients, which may be related to the bacterial load in the pleural fluid. The sensitivities of LAM detection in the pleural fluid were higher in confirmed TP than in clinically diagnosed TP patients (39.8% vs. 19.4%), with a specificity of 94.0%. The LAM level in plasma showed no significant differences between confirmed TP and clinically diagnosed TP patients. These findings indicated that LAM detection could be used not only to test Mtb but also to reflect the bacterial load, especially the LAM level in pleural fluid, which needs to be further verified.

## 5. Conclusions

We provide novel LAM detection choices in the pleural fluid and plasma of patients with TP. We found that LAM levels were significantly higher in the pleural fluid and plasma of patients with TP than in those of patients with malignant pleural effusion or the plasma of non-TB. LAM levels in the pleural fluid sample were significantly higher in confirmed TP patients than in clinically diagnosed TP patients. Our studies preliminarily indicated that LAM detection has a certain auxiliary diagnostic value for TP, especially for clinically diagnosed TP, which may help to improve the diagnosis of extrapulmonary tuberculosis.

## Figures and Tables

**Table 1:** Sensitivities of paired antibodies to detect purified LAM.

	Detection Antibody				
Capture Antibody	BJRbL01-Bio	BJRbL03-Bio	BJRbL20-Bio	BJRbL52-Bio	BJRbL76-Bio
BJRbL01	0.1	0.1	0.1	0.1	1.0
BJRbL03	0.1	1.0	0.1	0.1	1.0
BJRbL20	0.1	1.0	0.1	0.1	1.0
BJRbL52	0.1	1.0	1.0	1.0	1.0
BJRbL76	0.1	0.1	0.1	0.1	10.0

Unit is ng/mL.

**Table 2:** Characteristics of the study participants.

Characteristics	TP (*n* = 160)	MPE (*n* = 50)	Non-TB (*n* = 30)
Gender			
Male % (no.)	69.4% (111)	50.0% (25)	56.7% (17)
Female % (no.)	30.6% (49)	50.0% (25)	43.3% (13)
Age, mean ± SD, years	36.42 ± 15.91	59.82 ± 12.64	36.03 ± 10.09
Mycobacterial culture test			
Culture-positive % (no.)	41.9% (67)	—	—
Culture-negative % (no.)	58.1% (93)	—	—
Xpert MTB/RIF assay			
Xpert Mtb positive % (no.)	41.9% (67)	—	—
Xpert Mtb negative % (no.)	58.1% (93)	—	—
Patient subgroups			
Confirmed TP	55.0% (88)	—	—
Clinically diagnosed TP	45.0% (72)	—	—

TP = tuberculous pleurisy; MPE = malignant pleural effusion; non-TB = non-tuberculosis; SD = standard deviation.

## Data Availability

The data used to support the findings of this study are included within the article and are available from the corresponding author upon request.
